# Use of *In Vitro* and Predictive *In Silico* Models to Study the Inhibition of Cytochrome P4503A by Stilbenes

**DOI:** 10.1371/journal.pone.0141061

**Published:** 2015-10-20

**Authors:** Loai Basheer, Keren Schultz, Merav Fichman, Zohar Kerem

**Affiliations:** Institute of Biochemistry, Food Science and Nutrition, The Robert H. Smith Faculty of Agriculture, Food and Environment, The Hebrew University of Jerusalem, Rehovot, Israel; Weizmann Institute of Science, ISRAEL

## Abstract

CYP3A4 is recognized as the main enzyme involved in the metabolism of drugs and xenobiotics in the human body and its inhibition may lead to undesirable consequences. Stilbenes, including resveratrol, belong to a group of dietary health-promoting compounds that also act as inhibitors of CYP3A4. The aim of this study was to examine the use of computer modeling of enzyme-ligand interactions to analyze and predict the inhibition of structurally related compounds. To this end, an aldehyde group was attached to resveratrol and the interactions of CYP3A4 with resveratrol, its aldehyde analogue (RA) and a known synthetic inhibitor were studied and compared in two biological models. Specifically, the metabolism of testosterone was examined in a human intestine cell line (Caco-2/TC7) and in rat liver microsomes (RLM). The results demonstrated a weak inhibitory effect of RA on CYP3A4, as compared to resveratrol itself, in both biological models. Human CYP3A4 was more susceptible to inhibition than the commonly used model isozyme from rat. Modeling of the binding site of CYP3A4 revealed a combination of three types of interactions: hydrophobic interactions, electrostatic interactions and hydrogen bonds. A docking simulation revealed that the RA lacked an important binding feature, as compared to resveratrol, and that that difference may be responsible for its lower level of affinity for CYP3A4. Software analysis of binding affinity may serve as a predictive tool for designing new therapeutic compounds in terms of inhibition of CYP3A4 and help to reveal the biochemical nature of the interactions of dietary compounds, herbal compounds and drugs whose metabolism is mediated by this enzyme.

## Introduction

The cytochrome P450 enzymes (P450s) are responsible for the metabolism of a wide range of endogenous compounds (e.g., steroid hormones, lipids, bile acids), as well as xenobiotics including drugs, environmental pollutants and dietary products [1–3]. Of this large family of oxidizing enzymes, CYP3A4 is known to be the main enzyme involved in drug metabolism; it is involved in the metabolism of over 50% of all marketed drugs that rely on metabolic elimination [4, 5]. In light of this, the potential interactions between new molecules and CYP3A4 are assessed during the early stages of drug development [6, 7]. CYP3A4 is most abundant in the human liver, accounting for 30% of the total CYP protein content, but is also expressed in the gut, small intestine, prostate, breast and brain [5, 8–10]. The active site of a substrate-free cytochrome P450 contains a heme group as the fifth proximal ligand of the conserved cysteine and a water molecule as the sixth distal ligand [3]. Like most other CYPs, it catalyzes a monooxygenase reaction (i.e., the insertion of one atom of oxygen into an organic substrate while another oxygen atom is reduced to water) [11]. The substrate chemical characteristics and the preferred position of insertion vary from one CYP to another and are used to define the activity of a particular CYP in complex cellular systems [1, 12–14]. The presence of wild type CYP3A4 in the TC7 clone of Caco-2 cells is well established [15, 16], and allows the in vitro study of the enzyme’s modes of action and inhibition. Additional in vitro models are commonly used to further explore the uniformity of results achieved in the TC7 cell model, of which rat liver microsomes (RLM) are abundant [5, 17]. In both models, the levels of CYP3A activity are high and pre-defined, omitting potential regulation and expression effects.

Resveratrol (trans-3,4′,5-trihydroxystilbene) is a polyphenol found in grape skin and red wine. The inhibitory effects of resveratrol against CYP3A4 in vitro and in vivo are well established and it has been suggested that resveratrol acts as an irreversible, mechanism-based inactivator of this enzyme [18–22]. This mechanism-based inhibition occurs when a CYP3A4 substrate/inhibitor forms a reactive intermediate at the active site of CYP3A4, leading to enzyme inactivation as a result of modification to the heme or the apoprotein [23]. Chan and Delucchi suggested that an electron-rich unsaturated molecule like resveratrol could be a substrate for CYP3A4, where hydroxylation and epoxidation of resveratrol may occur, resulting in a reactive p-benzoquinone methide metabolite that is capable of binding covalently to CYP3A4, leading to inactivation during the course of catalysis [18]. Resveratrol exhibits a high level of membrane permeability and is categorized as a class-II compound in the Biopharmaceutical Classification System (BCS) [24]. However, resveratrol has a low bioavailability (less than 1%) due to the low water solubility (a logP value of 3.1), and the extensive first-pass metabolism in the intestine and in the liver, which extended by the enterohepatic recirculation. Further metabolism leads to the formation of the glucuronide and the sulfate metabolites of *t*-resveratrol [25, 26]. Recently, it was reported that resveratrol sulfates are deconjugated by steroid sulfatase to yield free resveratrol (in vitro and in vivo) and hence act as an intracellular reservoir for resveratrol [27]. Clinical and rat trials have found that the administration of resveratrol increases the area under curve (AUC) for several different drugs [21, 28]. Thus, high doses of resveratrol could theoretically increase the bioavailability and the risk of toxicity of drugs that undergo extensive first-pass metabolism by CYP3A4 [22].

**Fig 1:**
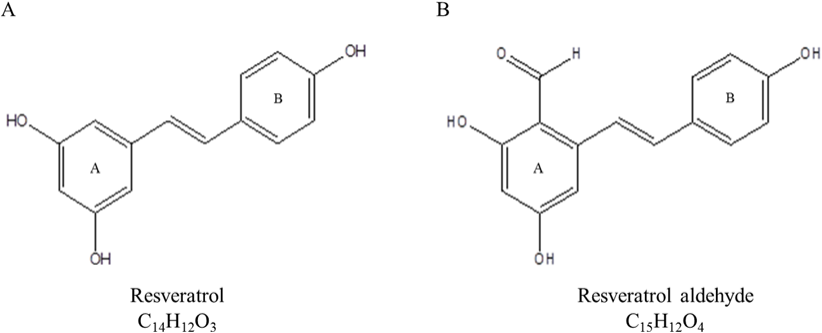
Chemical structures of resveratrol (A) and resveratrol aldehyde (B).

A study of the influence of lipophilicity on the interactions of hydroxyl stilbenes with CYP3A4 revealed that methoxy-stilbenes had lower IC_50_ values and greater affinity for CYP3A4 than the parent resveratrol and its glucosides [20]. The positive correlation between a molecule’s lipophilicity and its potential interaction with CYP3A4 has been further supported by QSAR studies [29–31]. In addition, the importance of hydrogen bonding in the interaction with CYP3A4 has been mentioned in several works [32–35]. A three-dimensional pharmacophore based on 38 substrates of CYP3A4 possessed two hydrogen bond (H-bond) acceptors, one H-bond donor, and one hydrophobic region [34]. The parent molecule of resveratrol contains three hydroxyl group (OH), which may act simultaneously as both H-bond donors, due to the hydrogen atom, and H-bond acceptors, due to the oxygen atom [36, 37]. However, the phenolic hydroxyl group is considered to be a strong H-bond donor and weak H-bond acceptor [38–40]. The oxygen atom of the aldehyde group acts as a strong H-bond acceptor [40]. Therefore, we assume that the addition of aldehyde group at position 2 on the A ring of resveratrol may contribute an H-bond acceptor moiety to the molecule and enhance its affinity for CYP3A4 (Fig 1). Huang and co-workers showed that RA exhibits a stronger inhibitory effect against xanthine oxidase and stronger anti-tumor activities than resveratrol and other analogues that have aldehyde groups [41]. The aim of this work was to study the effect of resveratrol and semi-synthesized resveratrol aldehyde (RA) on CYP3A4 activity in two in vitro models, a human cell culture (Caco2/TC7) and rat liver microsomes (RLM), and to examine the correlation between the biochemical findings with in silico results obtained through a virtual docking study. Examples illustrating the usefulness of simulation methods to understand protein-ligand interactions have been reported in a detailed review [42]. To the best of our knowledge, there have been no previous reports of studies of interactions between stilbenes and CYP3A4 that have been based on computational modeling and the comparison of the results of such modeling with the results of an in vitro experiment.

## Materials and Methods

### Chemicals and Reagents

Resveratrol (trans-3,4′,5-trihydroxystilbene, 99%), testosterone (99%) and ketoconazole (98%) standards were purchased from Sigma Aldrich, Israel. Testosterone [1,2,6,7-^3^H(N)] was purchased from (Perkin Elmer, USA). All other chemicals and reagents were purchased from Sigma Aldrich, unless otherwise noted.

### Synthesis of resveratrol aldehyde

(2,4-dihydroxy-6-((*E*)-2-(4-hydroxyphenyl)) benzaldehyde):

Resveratrol aldehyde (RA) was synthesized as described by Huang and co-workers [41]. Freshly distilled POCl_3_ (0.6 ml, 6 mmol) was added drop by drop to a solution of resveratrol (912 mg, 4 mmole) and DMF (464 ml, 6 mmol) in 20 ml of MeCN that was sitting in an ice-water bath. The mixture was stirred for 1 h at room temperature. Then, the solution was added to a mixture of ice and water, and the yellow mixture was stirred at 40°C in a water bath and extracted with EtOAc (3 × 10 ml). The combined organic layers were washed with water, dried over Na_2_SO_4_, filtered and evaporated. Purification by flash chromatography yielded product **2** 162 mg (yield 16%). ^**1**^ **H NMR** (200 MHz, MeOD, ppm): δ 6.17 (d, J = 2.2 Hz, 1H), 6.56 (d, J = 2.2 Hz, 1H), 6.76–6.81 (m, 2H), 6.92–6.99 (m, 1H), 7.39–7.47 (m, 3H), 10.19 (s, 1H). ^**13**^ **C NMR** (200 MHz, MeOD, ppm): δ 102.3, 107.5, 116.6, 120.9, 125.9, 129.5, (CH), 135.9, 144.5, 147.0, 159.2, 168.9 (C), 194.3 (CHO). ^1^H NMR and ^13^C NMR spectra of RA were obtained with a 200-MHz Bruker NMR spectrometer (Bruker Biospin GmbH, Rheinstetten, Germany).

### Maintenance of the Caco-2/TC7 cells

Caco-2/TC7, a subclone of Caco-2, expresses a high level of CYP3A4 enzyme, which is well presented in the human intestine [15, 43, 44]. The TC7 cell line is commonly used to study CYP3A4-mediated metabolism [16, 45, 46]. The Caco-2/TC7 cells used in this study were a kind gift from Dr. M. Rousset (INSERM U505, Paris) [15]. Maintenance of the cell line during all phases of the experiment was carried out under aseptic conditions in Dulbecco's modified Eagle medium (DMEM) at 37 C with 5% CO_2_ and 95% air. The DMEM was supplemented with 20% fetal bovine serum, 100 units/ml penicillin (Biological Industries, Israel), 100 μg/ml streptomycin (Biological Industries), 1% non-essential amino acids solution (concentrated 100X; Biological Industries) and 1% L-glutamine solution (200 mM, Biological Industries). The medium was pre-warmed at 37 C prior to contact with the cells. The Caco-2/TC7 cells were used between passages 25 and 35 and grown in 75 cm^2^ flasks, and new flasks were seeded at a concentration of 3–4 × 10^4^ cells/cm^2^.

### Testosterone metabolism in Caco-2/TC7 cells

Caco-2/TC7 cells were seeded at 2–3 × 10^4^ cells/cm^2^ on 12-well plates. The medium was changed every second day, and the cells were allowed to grow and differentiate up to 14 days after reaching confluence. On the experiment day, testosterone, ketoconazole, resveratrol and RA were dissolved in methanol, sonicated for 10 min (Transsonic T420/H, Elma, Germany) and added to the treatment medium (maximum methanol concentration in the medium was 0.1%). Testosterone, which was used as a substrate of CYP3A4 enzyme, was added to each cell well of all the treatments at final concentration of 200 μM. The treatments in this study were as follows: control 1, which included only testosterone incubated with Caco-2/TC7 cells; control 2, which included a specific inhibitor of CYP3A4; and ketoconazole (10 μM). Other treatments included different concentrations of resveratrol and RA (2.5, 5, 10 μM). All treatments were carried out for three incubation periods 2, 4 and 6 h (in addition to time 0) and there were four replicates of each treatment × incubation time combination. At the end of incubation, the culture medium was transferred to Eppendorf vials, the cell were harvested by scraping after the addition of HPLC-grade methanol (200 μl) and then transferred to the Eppendorf vials. Then, 100 μl of HPLC-grade acetonitrile was added and the vials were shaken vigorously, centrifuged at 14,000 RPM for 5 min (SIGMA Laborzentrifugen, Germany) and the supernatant was filtered through a 0.22 μm filter into HPLC vials.

### HPLC analysis

High pressure liquid chromatography (HPLC) was carried out on a Surveyor HPLC system (Thermo Finnigan, USA) using a RP-C18 Luna column 250 mm × 4.6 mm × 5 μm (Phenomenex, USA). Elution was performed with water (solvent A) and acetonitrile (solvent B) at a flow rate of 0.7 ml/min. The mobile phase gradient was modified as follow: B in A (v/v), from 5%-25% in 5 min, from 25%-75% in 10 min, from 75%-95% in 5 min followed by holding for 2 min, dropped again to 5% in 1 min and held for 4 min. The analytes were monitored with a photo diode array detector at 244 nm for testosterone and its metabolite, and at 280 nm and 306 nm for resveratrol and its derivatives. Peaks were scanned between 196 and 450 nm.

### Cell viability assay

The effects of resveratrol and RA on Caco-2/TC7 cells were examined by MTT assay. Cells were seeded onto 96-well plates at a density of 10^4^ cells/well and incubated for 24 h. Then, the cells were treated with DMEM (control) and resveratrol / RA (5, 10, 20, 50, 100 μM) for additional 8 h. Cells were incubated with 3-(4,5- dimethylthiazol-2-yl)-2,5-diphenyl tetrazolium bromide (MTT, 0.5 mg/ml) for 1 h and then with DMSO for 20 min. The formation of the colored formazan dye was assessed colorimetrically at 550 nm in an ELX 808 Ultra microplate reader (BIO-TEK Instruments, London, UK) using KCJunior software (York, UK).

### Preparation of rat liver microsomes

Male rat liver microsomes (RLM) were prepared from adult male Sprague-Dawley rats (175–199 g) according to published protocol with minor modifications [47]. Five livers were harvested from rats using a procedure approved by the Hebrew University Policy on Animal Care and Use, and the UFAW Handbook on the Care and Management of Laboratory Animals. Livers were weighed, frozen in liquid nitrogen, minced, and then stored at -80°C until use. Upon usage, minced livers were perfused with ice-cold homogenization buffer (50 mM Tris, pH 7.5, 150 mM KCl) at a ratio of 5 ml homogenization buffer per 1 g liver. Phenyl methyl sulfonyl fluoride (1 mM) was added right before homogenization. Livers were homogenized using a motorized homogenizer. The homogenate was centrifuged at 10,000 g for 10 min at 4°C. The supernatant was collected and centrifuged again at 100,000 g for 70 min at 4°C to yield microsomal pellets. Microsomes were re-suspended in ice-cold washing buffer (100 mM tetra-sodium pyrophosphate and 10 mM EDTA, pH 8.8) and re-pelleted by centrifugation at 100,000 g for 70 min at 4°C. The microsomal pellets were then re-suspended in ice-cold Tris buffer (50 mM, pH 7.5) containing 50% glycerol, transferred to vials (0.2 ml per vial) and stored at -80°C until use. Microsomal protein concentration was determined using the Bradford protein assay [48], in which a calibration of BSA (bovine serum albumin) was performed.

### Testosterone metabolism by microsomal CYP3A

The interaction of CYP3A4 with testosterone was examined using the tritiated water assay adapted from González and Piferrer [49]. Reactions were carried out in 96-well plates (Nunc, Roskilde, Denmark) and all samples were assayed in triplicate. RLM were thawed in ice and 100 μg was added to each well with reaction buffer (10 mM K_2_HPO_4_, 100 mM KCl, 1 mM EDTA, 1 mM dithiothreitol, pH 7.4) containing 8 nM testosterone [1, 2, 6, 7-^3^H (N)] and 1.5 mM NADPH. Resveratrol, RA and ketoconazole were first dissolved in 30% methanol and sonicated, then stocks solutions were prepared in reaction buffer (maximum methanol concentration in each well was 0.95%). The different treatments were added to the wells to achieve final concentrations of 2, 5, 10, 25, 50, 75 and 100 μM for resveratrol and RA, and 2.5, 25, 50, 100 and 200 μM for ketoconazole. All treatments were assayed in triplicate with the following controls: a buffer control containing the reaction buffer instead of the inhibitor (no treatment), a methanol control containing 0.95% methanol instead of the inhibitor (to account for possible effects of this solvent) and an RLM control without any microsomes, to provide the background value. Reactions were then incubated for 4 h with continuous shaking at the optimal temperature of 37°C. The addition of 1 ml of ethyl ether followed by vortex mixing was used to stop the reactions. Ether and aqueous layers were allowed to separate for 10 min, and then transferred to -80°C for 15 minutes for a complete freeze of the aqueous phase. The aqueous phase was left for 1 h in the chemical hood for a complete evaporation of ether traces. The aqueous phase was shacked with 2 volumes (400 μl) of dextran coated (0.5%) charcoal slurry (5% coated charcoal in phosphate buffer) for 1 min and centrifuged at 3000 g at 4°C for 5 min. The supernatant (300 μl) was collected into scintillation vials and mixed with 5 ml scintillation fluid. ^3^H was measured as disintegration per 5 min using a liquid scintillation β counter (Canberra-Packard Tri-Crab 1600, CA).

### Computational modeling and simulation software

The crystal structure of human CYP3A4 was determined with ketoconazole [50], available in the protein data bank (PDB entry 2V0M). Sketches of the structures of the ligands and logP values were obtained using ChemDraw Ultra 8.0 and energy minimization of ligands was achieved with Chem3D Ultra 8.0 (CambridgeSoft Corporation, USA). Ligands in a MDL molfile were uploaded into Discovery Studio 4.0 (Accelrys, San Diego, CA) and the “clean geometry” tool was used to minimize the structure energy. Conformations were then generated using “generate conformations” protocol using “Best” method (other parameters were set at their default values). 2V0M represents a tetrameric crystal structure of CYP3A4 that includes four chains (A-D). Isotropic displacements were colored to show temperature values of atoms and demonstrate the uncertainty in the positions of atoms in the proposed structure [51]. Chain D showed the lowest temperature values, indicating the highest confidence in its atoms positions, and hence selected for the simulations. The second molecule of ketoconazole, which was identified in an antiparallel orientation above the first ketoconazole molecule, was removed, along with the extracellular and intracellular loops, since they are predicted to not be part of the binding site. Prior to the analysis, the iron atom in the heme group was constrained to preserve its bonding to the nitrogen atoms of the heme after the application of the CHARMm force field. The protein was prepared using the “prepare protein” protocol and a binding site was created using the “define and edit binding site” protocol (default parameters were used). Each ligand conformation that had been previously generated was superimposed on ketoconazole from the 2V0M, according to the alignment specification (80% steric and 20% electrostatic) and the top five conformations with the highest degree of similarity to ketoconazole were chosen for the docking simulations. Docking of the ligands (ketoconazole, resveratrol and RA) was performed using “CDOCKER” protocol. After the docking, analysis of the ligands’ interactions at the binding site was performed using the “ligand interactions” tool and the final analysis was performed manually. All protocols and tools are available in Discovery Studio 4.0, Accelrys.

## Results

### Testosterone metabolism in the Caco-2/TC7 cell line

**Fig 2:**
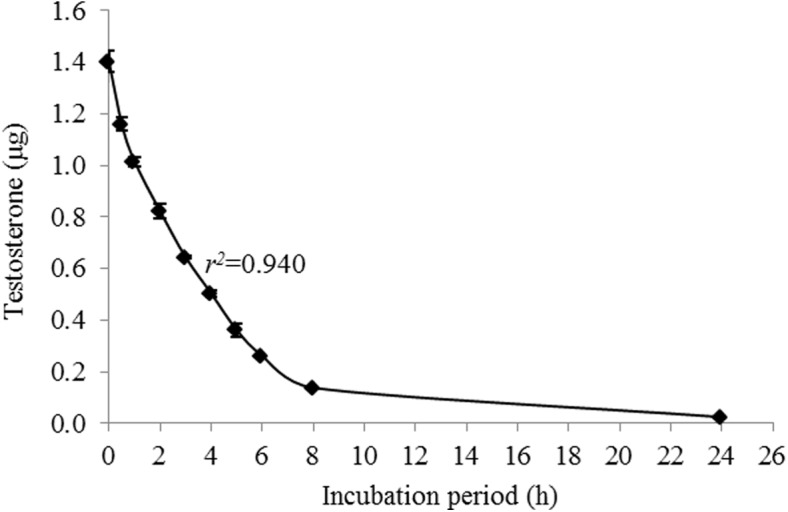
Metabolism of testosterone in the Caco-2/TC7 cell line. Data are mean ± S.E. values of three replicates.

Testosterone metabolism mediated by CYP3A4 was assessed based on the quantification of testosterone retained in the medium after incubation with Caco-2/TC7 cells. Testosterone metabolism was initially followed over a 24-h incubation period (Fig 2) and a 6-h incubation was chosen for further experiments. Testosterone was quantified using HPLC and a calibration curve.

### Effects of resveratrol and resveratrol aldehyde on cell viability

**Fig 3:**
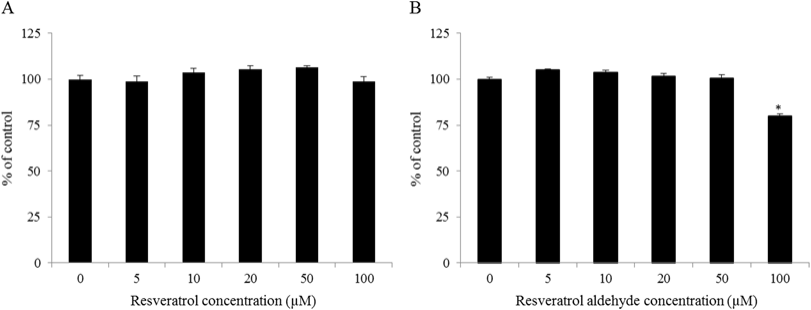
Caco-2/TC7 cell viability when different concentrations of resveratrol (A) or resveratrol aldehyde (B) were present in the medium. Data are mean ± S.E. values of five replicates. Star indicates significant differences at *P*< 0.05 as determined by ANOVA, followed by the Tukey-Kramer test.

The effects of resveratrol and RA on the viability of Caco-2/TC7 cells were measured as described. Those measurements revealed that resveratrol did not affect the cells’ viability within the examined concentration range, but that amending the medium with 100 μM RA did reduce cell viability. None of the other examined concentrations of RA reduced cell viability (Fig 3). Thus, 50 μM was selected as the highest concentration to be applied to Caco-2/TC7 cells.

### Inhibition of CYP3A4-mediated testosterone metabolism in the Caco-2/TC7 cell line by resveratrol and resveratrol aldehyde

**Fig 4:**
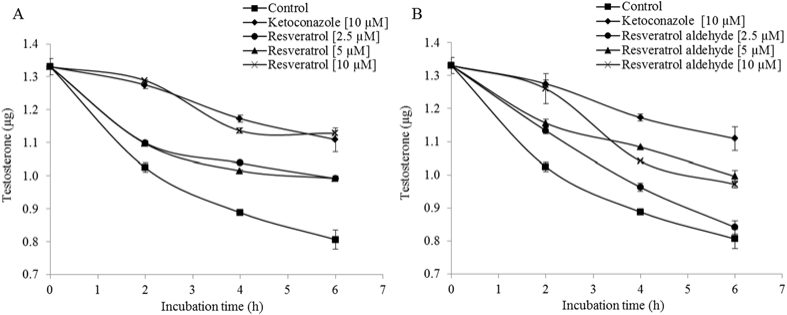
Inhibition of testosterone metabolism in Caco2/TC7 cell line by different concentrations of resveratrol (A) and resveratrol aldehyde (B) for different incubation periods, as compared to the control (no inhibition) and the ketoconazole treatment. Data are mean ± S.E. values of three replicates.

**Fig 5:**
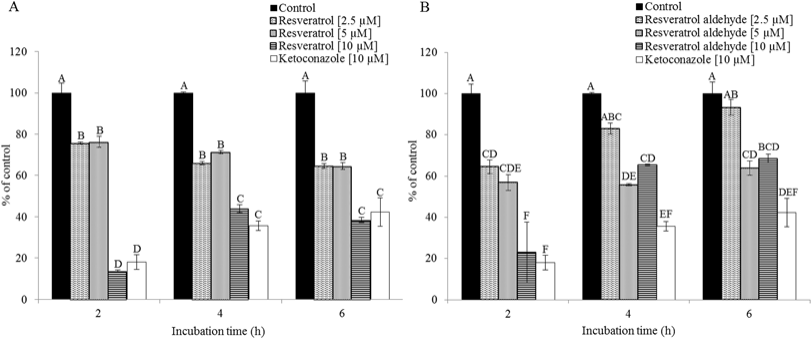
Inhibition of testosterone metabolism in Caco2/TC7 cells by different concentrations of resveratrol (A) and resveratrol aldehyde (B) for different incubation periods, as compared to the control (no inhibition) and the ketoconazole treatment. Results are expressed as the percentage of the activity of the control for each incubation period. Data are mean ± S.E. values of three replicates. Different letters indicate significant differences at *P* < 0.05 as determined by ANOVA, followed by the Tukey-Kramer test.

CYP3A4-mediated metabolism of testosterone in Caco-2/TC7 cell was recorded in 2-h intervals and during 6-h periods, based on the pattern shown above (Fig 2). Both resveratrol and RA were shown to inhibit this metabolism (Figs 4 and 5): All resveratrol treatments were significantly different from the control for all of the examined incubation periods and strong inhibition, similar to that of ketoconazole, was observed at 10 μM. Moderate inhibition was observed at lower concentrations of resveratrol (Figs 4A and 5A). Similarly, RA inhibited the enzymatic oxidation of testosterone in a dose-dependent manner, especially during the first 2 h of incubation. Later, the inhibition continued less intensely. However, the inhibition of the enzyme in the presence of 5 and 10 μM of RA were significant for all of the examined incubation periods and in the presence of 2.5 μM for the first 4 h of incubation (Figs 4B and 5B). The loss of inhibition at the lower concentration of RA may suggest a competitive, rather than suicidal mode of inhibition.

### Inhibition of microsomal-CYP3A activity by resveratrol and resveratrol aldehyde

**Fig 6:**
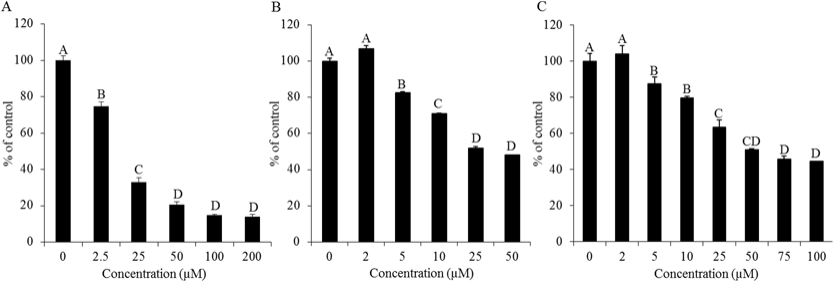
Inhibition of CYP3A-mediated formation of tritiated water. **Reduction in microsomal CYP3A activity at different concentrations of ketoconazole (A), resveratrol (B) and resveratrol aldehyde (C).** The rate of reduction was calculated by setting the control equal to 100%. Data are mean ± S.E. values of three replicates. Different letters indicate significant differences at *P* < 0.05 as determined by ANOVA, followed by the Tukey-Kramer test.

All of the tested compounds inhibited the formation of tritiated water following the metabolism of testosterone [1,2,6,7-^3^H(N)] mediated by CYP3A in the RLM. The inhibition rate is expressed as a percentage relative to that of the control. The ketoconazole treatment represents the highest inhibition capacity (Fig 6A), with an IC_50_ value of 14 μM (Table 1). Resveratrol and RA had significant inhibitory effects starting from the concentration of 5 μM and the inhibitory effects of these two substances proceeded in a similar manner (Fig 6B and 6C). However, resveratrol had a higher inhibition capacity than RA, with IC_50_ values of 28 μM and 51 μM, respectively (Table 1). The 50% inhibition concentration of RA was not found within the range of concentrations examined in the TC7 assay. Therefore, the IC_50_ values of the tested compounds are presented in a uniform manner based on the results of the RLM assay.

**Table 1:** IC_50_ values of microsomal CYP3A inhibition.

Compound	IC_50_ (μM)
Ketoconazole	14
Resveratrol	28
Resveratrol aldehyde	51

Values were graphically estimated from concentration–response curves.

### Computational analysis

**Fig 7:**
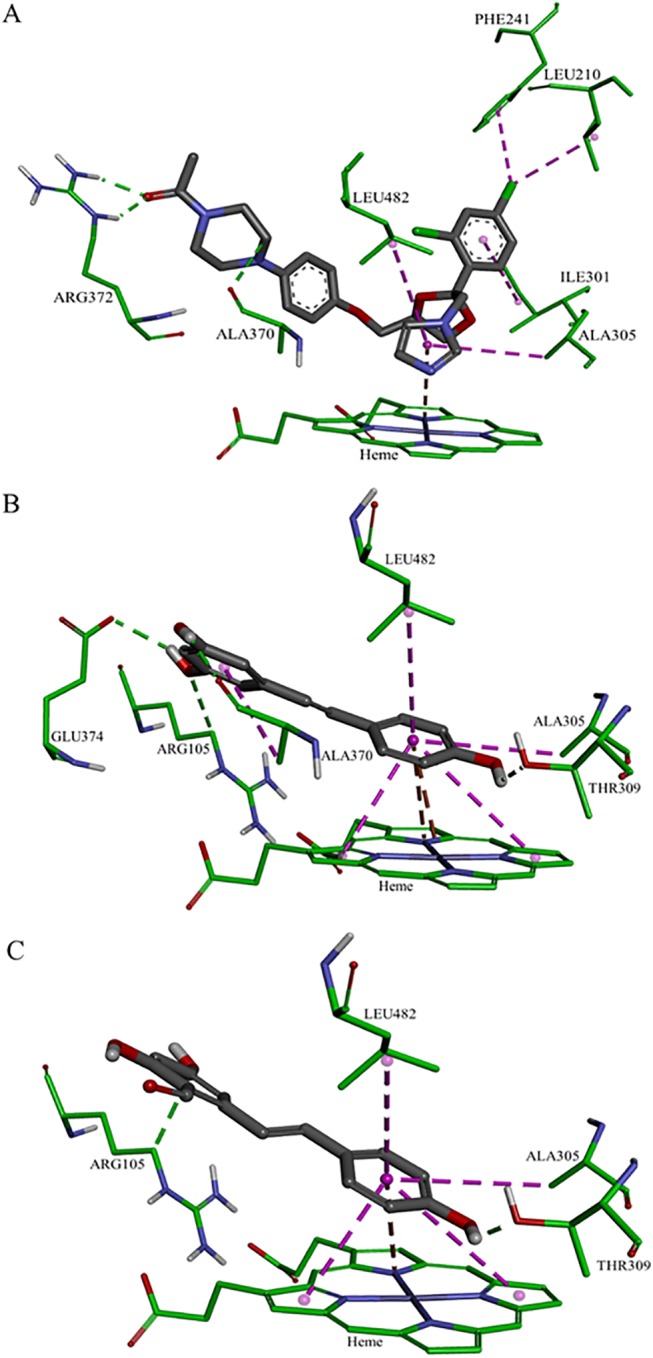
Docking of the ligands at the binding site of CYP3A4, showing the interacting residues: ketoconazole (A), resveratrol (B) and resveratrol aldehyde (C). Ligands are shown as gray sticks; receptor residues are shown as green sticks. Bonds are shown with dashed lines colored as follows: hydrophobic interactions in magenta, electrostatic interaction in brown and hydrogen bonds in green.

**Fig 8:**
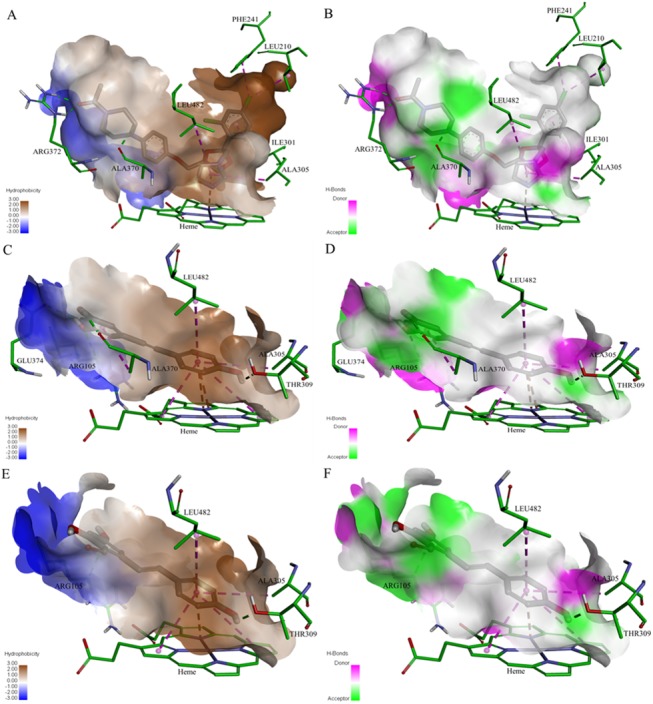
The binding site of CYP3A4 docked with the different ligands, showing the maps of hydrophobicity (left) and hydrogen bonds (right): ketoconazole (A-B), resveratrol (C-D) and resveratrol aldehyde (E-F). Ligands are shown as gray sticks; receptor residues are shown as green sticks.

The orientation and residue bonding of the ketoconazole molecule inferred from the X-ray structure of human CYP3A4 (PDB code 2V0M) served as a model for that of resveratrol and RA. In order to validate the suitability of the selected docking model, we first docked ketoconazole and ensured that its bonding to the CYP3A4 binding site was restored to the initial state, as in the original 2V0M structure. According to the ketoconazole docking simulation, the binding site can be thought of in terms of three main features: a solid hydrophobic region, H-bond donors/acceptors and electrostatic interactions. The hydrophobic cluster includes the alkyl and alkyl-pi interactions between CYP3A4 residues Leu210, Phe241, Ile301, Ala305 and Leu482 and the chlorinated aromatic ring and imidazole ring of the ketoconazole molecule. The main H-bond donor residue is CYP3A4 Arg372, which interacts with the ketonic oxygen atom of ketoconazole; whereas the oxygen atoms of Ala370 serve as H-bond acceptors. The nitrogen atom of the heme group provides further anchoring to the molecule through its electrostatic interactions (anion-pi) with the imidazole ring of the ketoconazole (Figs 7A and 8A and 8B). A resveratrol molecule interacts with the enzyme somewhat like ketoconazole does, but is also involved in other interactions at the binding site. Since resveratrol is shorter than ketoconazole, it is incapable of carrying out a significant part of the hydrophobic interactions, such as those between ketoconazole and residues Leu210, Phe241 and Ile301. On the other hand, the B ring of resveratrol is able to engage in additional hydrophobic and electrostatic interactions at the binding site. There are alkyl-pi interactions between the B ring of resveratrol and residues Ala305 and Leu482, as well as stacked pi–pi interactions with two pyrrole rings of the heme group; whereas cation-pi and anion-pi interactions were observed with the heme iron and nitrogen, respectively. There are additional hydrophobic interaction between the A ring and Ala370. The hydrogen atoms of the three hydroxyl groups of resveratrol act as H-bond donors and bind Ala370 and Glu374 (ring A), and Thr309 (ring B; Figs 7B and 8C and 8D). RA docking represents most of the resveratrol interaction, aside from the cation-pi interaction with the heme iron, H-bonding to Glu374 and the interactions with Ala370 (Figs 7C and 8E and 8F). According to the observed docking energy values, ketoconazole has the strongest affinity for CYP3A4 with the lowest docking energy value; resveratrol and RA had the next highest docking energies (Table 2).

**Table 2:** CDOCKER energy values of the ligands into CYP3A4.

Compound	CDOCKER energy (kcal/mole)
Ketoconazole	-40.752
Resveratrol	-25.504
Resveratrol aldehyde	-17.717

## Discussion

Among the members of the P450 enzyme family, CYP3A4 is recognized as the main enzyme involved in the metabolism of drugs in the liver and, no less importantly, in the gut [5]. Potential interactions between promising new drugs and CYP3A4 are assessed during the earliest stages of drug development [6, 8, 52]. In recent studies, evidence has accumulated to indicate potent interactions between CYP3A4 and dietary phytochemicals [5]. These compounds, some of which are abundant in our diet, belong to the large and diverse family of polyphenolics, which includes flavonoids, phenolic acids, phenolic alcohol, stilbenoids and lignans [5, 53, 54]. The inhibitory effects of *t*-resveratrol on CYP3A4, both in vitro and in vivo, are well documented in the literature [18–22] and clinical trials have found that the administration of resveratrol increases the plasma concentrations of several drugs [22, 28]. A previous study of the effect of a molecule’s lipophilicity on its interactions with CYP3A4 using synthetic modifications of resveratrol revealed that methoxy-stilbenes have lower IC_50_ values and greater affinity for CYP3A4, as compared to the parent resveratrol and its glucosides [20]. QSAR and pharmacophore mapping studies also point to the importance of lipophilicity, as well as the role of hydrogen bonding in determining how various molecules interact with CYP3A4 [29, 30, 32, 33, 35, 55]. Here, the structure–activity relationships of resveratrol in the inhibition of the metabolism of testosterone by CYP3A4 were studied using two different biological models and simulation software.

Results of the examination of the inhibition of testosterone metabolism in the Caco-2/TC7 cells line reveal inhibitory effects of both resveratrol and RA on CYP3A4. The residence time of substances from ingested foods, drugs and others is estimated to be 1.5–2 h in the Jejunum and 5–7 h in the Ileum [56]. The metabolism of testosterone using Caco-2/TC7 cells was designed to last 8 h to optimally represent passage in the intestine. A linear curve was observed from 2 to 6 h incubation period, leading to the selection of this time interval to study the inhibition of human intestinal CYP3A4. We monitored the substrate consumption methodology and, in this context, the treatments were significantly different from the control throughout all of the examined incubation periods. As demonstrated, the inhibition capacity of resveratrol was found to be higher than that of its aldehyde analogue. 10 μM of resveratrol inhibited CYP3A4 in a pattern similar to that of ketoconazole, which was used as a positive control; in the 10 μM resveratrol treatment, enzyme activity decreased to less than 50%, of that observed for the control. RA at the same concentration had a similar effect during the first 2 h of the incubation period, but that inhibition decreased after that time point. The treatments of 2.5 μM and 5 μM of resveratrol and RA inhibited CYP3A4 activity in 30% (on average) throughout the incubation periods. These results from the assay in the human intestinal cell line are consistent with the findings from the assay carried out using RLM, in which the IC_50_ value of resveratrol (28 μM) was found to be lower than that of RA (51 μM) and ketoconazole exhibited the strongest inhibitory effect (IC_50_ = 14 μM). The IC_50_ value of resveratrol for CYP3A falls in the range found in the literature for RLM models [19, 57] and similar-scale IC_50_ values of ketoconazole (3.7–13 μM) have been reported previously [17, 58, 59]. Further comparison between the results of the two models, TC7 cells and RLM, revealed that CYP3A4 inhibition appears to be more sensitive in the human intestinal cell line than in rat liver microsomes. In the TC7 cells, 10 μM of ketoconazole or resveratrol was enough to inhibit CYP3A4 by more than half; whereas in the RLM, the IC_50_ values were 14 μM for ketoconazole and 28 μM for resveratrol. Piver et al. reported a similar conclusion when the resveratrol IC_50_ value for CYP3A-mediated testosterone 6β-hydroxylation in RLM was observed to be much higher than that in human liver microsomes (20 versus 4 μM) [19]. Further indications of the higher susceptibility of human CYP3A4 compared to the rat isozyme can be found in the literature [60–62].

There are no remarkable differences between the logP values of ketoconazole, resveratrol and RA (Table 3). However, the addition of an aldehyde group at position 2 on the A ring of resveratrol slightly decreased the logP value as compared to that of the parent molecule (2.80 versus 3.06). Previous work conducted in our laboratory revealed an inverse correlation between the values of the experimental partition coefficients and the IC_50_ values for CYP3A4 and resveratrol and its analogs. In that work, methoxy-stilbenes, resveratrol and resveratroloside were found to have partition coefficients of 2.51, 1.87 and 0.89 and IC_50_ values of 0.47 μM, 1.4 μM and >40 μM, respectively [20, 63].

**Table 3:** LogP values of the compounds.

Compound	LogP
Ketoconazole	3.54
Resveratrol	3.06
Resveratrol aldehyde	2.80

The results of the present work lead us to a similar conclusion regarding the inhibitory effects of the tested compounds, expressed in IC_50_ values and CDOCKER energies, which were found to be in reverse order to their logP values. In line with the logP values, ketoconazole had the highest lipophilicity and the highest number of hydrophobic interactions in the binding site of CYP3A4 (Fig 7A). Some of those interactions were not observed for resveratrol and RA, especially those that involve the chlorinated aromatic ring of ketoconazole (Leu210, Phe241 and Ile301; Fig 7C and 7E). Hence, this work confirms the well-known need of the molecule for a solid hydrophobic region to interact with CYP3A4 [29–32, 34, 55]. On the other hand, resveratrol compensates with other interactions due to the position of the B ring in relation to the heme group, which allows for hydrophobic (stacked pi–pi) and electrostatic interactions with the pyrrole rings and the iron/nitrogen atoms of the heme. The role of the heme-iron in the metabolic mechanism of CYP enzymes is well established [64–66]. The three hydroxyl groups of resveratrol have conventional H-bonds; the hydrogens in positions 3, 5 and 4’ act as donors to the oxygen of the residues Ala370, Glu374 and Thr309, respectively. Although RA also has three hydroxyl groups, only its 4’ hydroxyl group forms a conventional H-bond with Thr309; whereas the hydroxyl groups at positions 3 and 5 do not interact with the binding site. We suggest that steric repulsions might shift the A ring hydroxyl groups some distance away from H-bond acceptors at the binding site, thereby reducing the affinity of RA for CYP3A4, as compared to the parent molecule.

Taken together, according to the above results, the interactions at the CYP3A4 binding site can be categorized into three groups: hydrophobic, electrostatic and H-bond interactions. Despite the fact that it does not engage in all of the hydrophobic interactions that ketoconazole does, resveratrol still engages in the main interactions in these three categories. RA represents a decrease in the intensity of some important interactions, especially those involving the heme-iron and H-bonding at the A ring of the molecule, which explains its lower level of affinity and consequently less severe inhibitory effect on CYP3A4.

## Conclusions

Here, we report for the first time on an inhibitory effect of RA, a semi-synthesized analogue of resveratrol, on CYP3A4 in human cell line and CYP3A in rat liver microsomes. The predictive power of computational modeling for the study of the interactions of CYP enzymes with candidate health-promoting compounds is demonstrated. We report a lower inhibitory capacity for RA as compared to the closely related resveratrol, which can be attributed to RA engaging in fewer hydrophobic and electrostatic interactions at the binding site of CYP3A4, as calculated using docking modeling. We suggest that the use of computational tools (e.g. docking in particular) may prove to be quite useful for predicting CYP3A4 inhibition by candidate therapeutics, as well as interactions between consumed phytochemicals and prescribed drugs. Moreover, integration of results from various enzymatic models and computer modeling of docking promotes comprehensive understanding of these interactions.

## Supporting Information

S1 File Testosterone metabolism in Caco2/TC7 cells. (PDF)

S2 File Testosterone metabolism in RLM. (PDF)
