# Supplementary material for: Use of In Vitro and Predictive In Silico Models to Study the Inhibition of Cytochrome P4503A by Stilbenes
Source: PLoS One. 2015 Oct 20;10(10):e0141061. doi: 10.1371/journal.pone.0141061 (PMC4618141; doi:10.1371/journal.pone.0141061)
Supplement: S1 File — (PDF) [file pone.0141061.s001.pdf]

# Supplementary file PONE-D-15-21369

## S1. Testosterone metabolism in Caco-2/TC7 cells (CYP3A4)

Testosterone quantified based on HPLC-calibration

| | | Testosterone (µg) | | | | Averages | | | | S.E. | | | |
|---|---|---|---|---|---|---|---|---|---|---|---|---|---|
| Treatment | Repeat | Incubation time (h) | | | | Incubation time (h) | | | | Incubation time (h) | | | |
| | | 0 | 2 | 4 | 6 | 0 | 2 | 4 | 6 | 0 | 2 | 4 | 6 |
| Control | 1 | 1.331 | 0.996 | 0.892 | 0.831 | 1.330 | 1.024 | 0.888 | 0.806 | 0.025 | 0.014 | 0.002 | 0.030 |
| | 2 | 1.287 | 1.042 | 0.886 | 0.840 | | | | | | | | |
| | 3 | 1.374 | 1.034 | 0.886 | 0.747 | | | | | | | | |
| Ketoconazole [10 µM] | 1 | 1.331 | 1.293 | 1.157 | 1.047 | 1.330 | 1.275 | 1.173 | 1.109 | 0.025 | 0.011 | 0.010 | 0.036 |
| | 2 | 1.287 | 1.255 | 1.192 | 1.109 | | | | | | | | |
| | 3 | 1.374 | 1.278 | 1.168 | 1.171 | | | | | | | | |
| Resveratrol [2.5 µM] | 1 | 1.331 | 1.095 | 1.043 | 1.004 | 1.330 | 1.099 | 1.039 | 0.992 | 0.025 | 0.002 | 0.004 | 0.006 |
| | 2 | 1.287 | 1.101 | 1.031 | 0.984 | | | | | | | | |
| | 3 | 1.374 | 1.100 | 1.041 | 0.987 | | | | | | | | |
| Resveratrol [5 µM] | 1 | 1.331 | 1.104 | 1.015 | 1.009 | 1.330 | 1.097 | 1.015 | 0.992 | 0.025 | 0.008 | 0.003 | 0.009 |
| | 2 | 1.287 | 1.106 | 1.020 | 0.980 | | | | | | | | |
| | 3 | 1.374 | 1.080 | 1.009 | 0.987 | | | | | | | | |
| Resveratrol [10 µM] | 1 | 1.331 | 1.285 | 1.131 | 1.124 | 1.330 | 1.289 | 1.136 | 1.129 | 0.025 | 0.002 | 0.008 | 0.007 |
| | 2 | 1.287 | 1.289 | 1.125 | 1.120 | | | | | | | | |
| | 3 | 1.374 | 1.291 | 1.152 | 1.143 | | | | | | | | |

| | | Testosterone (µg) | | | | Averages | | | | S.E. | | | |
|---|---|---|---|---|---|---|---|---|---|---|---|---|---|
| Treatment | Repeat | Incubation time (h) | | | | Incubation time (h) | | | | Incubation time (h) | | | |
| | | 0 | 2 | 4 | 6 | 0 | 2 | 4 | 6 | 0 | 2 | 4 | 6 |
| Control | 1 | 1.331 | 0.996 | 0.892 | 0.831 | 1.330 | 1.024 | 0.888 | 0.806 | 0.025 | 0.014 | 0.002 | 0.030 |
| | 2 | 1.287 | 1.042 | 0.886 | 0.840 | | | | | | | | |
| | 3 | 1.374 | 1.034 | 0.886 | 0.747 | | | | | | | | |
| Ketoconazole [10 µM] | 1 | 1.331 | 1.293 | 1.157 | 1.047 | 1.330 | 1.275 | 1.173 | 1.109 | 0.025 | 0.011 | 0.010 | 0.036 |
| | 2 | 1.287 | 1.255 | 1.192 | 1.109 | | | | | | | | |
| | 3 | 1.374 | 1.278 | 1.168 | 1.171 | | | | | | | | |
| Resveratrol aldehyde (RA) [2.5 µM] | 1 | 1.331 | 1.115 | 0.948 | 0.881 | 1.330 | 1.133 | 0.963 | 0.841 | 0.025 | 0.010 | 0.012 | 0.020 |
| | 2 | 1.287 | 1.149 | 0.955 | 0.829 | | | | | | | | |
| | 3 | 1.374 | 1.135 | 0.986 | 0.814 | | | | | | | | |
| Resveratrol aldehyde (RA) [5 µM] | 1 | 1.331 | 1.165 | 1.087 | 1.020 | 1.330 | 1.156 | 1.084 | 0.996 | 0.025 | 0.012 | 0.002 | 0.018 |
| | 2 | 1.287 | 1.170 | 1.085 | 1.006 | | | | | | | | |
| | 3 | 1.374 | 1.133 | 1.079 | 0.961 | | | | | | | | |
| Resveratrol aldehyde (RA) [10 µM] | 1 | 1.331 | 1.206 | 1.045 | 0.982 | 1.330 | 1.260 | 1.041 | 0.972 | 0.025 | 0.045 | 0.002 | 0.011 |
| | 2 | 1.287 | 1.350 | 1.037 | 0.950 | | | | | | | | |
| | 3 | 1.374 | 1.225 | 1.041 | 0.983 | | | | | | | | |

| Res_Statistics- Tukey letters | | | |
|---|---|---|---|
| Treatment | Incubation time (h) | | |
| | 2 | 4 | 6 |
| Control | A | A | A |
| Resveratrol [2.5 µM] | B | B | B |
| Resveratrol [5 µM] | B | B | B |
| Resveratrol [10 µM] | D | C | C |
| Ketoconazole [10 µM] | D | C | C |

| RA_Statistics- Tukey letters | | | |
|---|---|---|---|
| Treatment | Incubation time (h) | | |
| | 2 | 4 | 6 |
| Control | A | A | A |
| Resveratrol aldehyde [2.5 µM] | CD | ABC | AB |
| Resveratrol aldehyde [5 µM] | CDE | DE | CD |
| Resveratrol aldehyde [10 µM] | F | CD | BCD |
| Ketoconazole [10 µM] | F | EF | DEF |
