# Supplementary material for: Use of In Vitro and Predictive In Silico Models to Study the Inhibition of Cytochrome P4503A by Stilbenes
Source: PLoS One. 2015 Oct 20;10(10):e0141061. doi: 10.1371/journal.pone.0141061 (PMC4618141; doi:10.1371/journal.pone.0141061)
Supplement: S2 File — (PDF) [file pone.0141061.s002.pdf]

<u>**Supplementary file PONE-D-15-21369**</u>

S2. Testosterone metabolism in Rat Liver Microsomes (CYP3A4)

Compound	Concentration (µM)	% of control	Average	S.E.
**Control**	0	103.2647217	100.00	2.54
	0	101.7325935		
	0	95.0026848		
**Ketoconazole**	2.5	73.87506712	74.82	2.42
		79.40934312		
		71.19026311		
	25	37.78414176	33.06	2.41
		31.52675855		
		29.88007875		
	50	21.99749418	20.49	1.75
		17.01449794		
		22.47001969		
	100	15.7615894	14.82	0.54
		14.82011813		
		13.87864686		
	200	16.17683909	13.90	1.45
		14.32253445		
		11.19384285		
**Resveratrol**	2	103.83	106.69	1.66
		106.64		
		109.60		
	5	78.64	82.46	1.96
		83.67		
		85.08		
	10	70.65	71.07	0.74
		70.05		
		72.51		
	25	51.28	51.76	0.26
		52.16		
		51.84		
	50	48.18	48.25	0.89
		46.75		
		49.82		

Keto- statistics					
Level	Tukey-letters				Mean
Control	A				100.00
Keto 2.5 µM		B			74.82
Keto 25 µM			C		33.06
Keto 50 µM				D	20.49
Keto 100 µM				D	14.82
Keto 200 µM				D	13.90

Res- statistics					
Level	Tukey-letters				Mean
Res 2 µM	A				106.69
Res 5 µM		B			82.46
Res 10 µM			C		71.07
Res 25 µM				D	51.76
Res 50 µM				D	48.25

**Resveratrol Aldehyde (RA)**	2	96.71	104.07	4.24
		104.10		
		111.41		
	5	86.46	87.50	4.47
		80.32		
		95.71		
	10	81.17	79.65	3.76
		85.26		
		72.51		
	25	63.34	63.48	0.70
		64.76		
		62.33		
	50	55.41	50.78	3.97
		54.05		
		42.89		
	75	46.06	45.71	0.67
		44.42		
		46.65		
	100	46.67	44.54	1.61
		45.57		
		41.39		

RA- statistics					
Level	Tukey-letters				Mean
RA 2 µM	A				104.07
RA 5 µm		B			87.50
RA 10 µM		B			79.65
RA 25 µM			C		63.48
RA 50 µM			C	D	50.78
RA 75 µM				D	45.71
RA 100 µM				D	44.54
